# Association of neck circumference and hypertension among adults in a rural community Thailand: A cross-sectional study

**DOI:** 10.1371/journal.pone.0256260

**Published:** 2021-08-20

**Authors:** Panuwat Soitong, Saowaluck Jangjaicharoen, Apisit Kaewsanit, Parinya Mali, Yada Viriyakhaikul, Supakarn Boonnumma, Suphat Tipmabutr, Paratakorn Chalermchuang, Warunporn Maneechot, Chanunchida Numnoi, Kitwiwat Phungmali, Thana Meksong, Benjapon Ponpadermyod, Wachara Jirachairattanasin, Boonsub Sakboonyarat, Ram Rangsin, Mathirut Mungthin, Phunlerd Piyaraj

**Affiliations:** 1 Fourth Year Medical Cadet, Phramongkutklao College of Medicine, Bangkok, Thailand; 2 Department of Military and Community Medicine, Phramongkutklao College of Medicine, Bangkok, Thailand; 3 Department of Pharmacology, Phramongkutklao College of Medicine, Bangkok, Thailand; 4 Department of Parasitology, Phramongkutklao College of Medicine, Bangkok, Thailand; Shanghai Institute of Hypertension, CHINA

## Abstract

**Introduction:**

Hypertension (HT) is a major non-communicable disease worldwide and a growing global public health problem. Although several studies have investigated the independent associations of neck circumference (NC) and hypertension, no such studies have been conducted among the Thai population.

**Aim:**

This study aims to identify risk factors associated with hypertension, which may be used to predict HT among asymptomatic adults residing in a remote rural community in central Thailand.

**Method:**

1,084 adults were included in this community-based cross-sectional study by a population-based total survey. The participants were included those who had been living in 6 villages in the rural community in the central area of Thailand. Anthropometric information, NC, body composition indexes such as waist circumference and blood pressure were measured. Logistic regression models were fitted to calculate the multi-variable adjusted prevalence and the association of NC with HT.

**Result:**

The prevalence of HT among adults in the rural community was 27.7% (95% CI: 25.0–30.3). Of the 300 adults with HT, 164 participants (54.7%) were found within the unawareness HT category. We found that associated factors with HT were included larger neck circumference both continuous and categorical (≥ 37.5 in male, ≥ 32.5 in female), pre-existing diabetes mellitus, male, and higher body mass index.

**Conclusion:**

Almost one-third of participants in the remote rural areas presented hypertension. NC was associated with HT independent from other risk factors. NC is a simple and useful anthropometric index to identify HT in rural Thai adults.

## Introduction

Hypertension (HT) is a major non-communicable disease worldwide. Moreover, it is confirmed that HT also leads to cardiovascular complications and deaths [1]. High blood pressure is associated with atherosclerotic cardiovascular diseases (ASCVD) including stroke, ischemic heart disease, and peripheral vascular disease [2, 3]. The number of adults with elevated blood pressure (BP) rose from 594 million in 1975 to 1.13 billion in 2015; 258 million of whom were living in South Asian countries [4]. The previous report of the 5^th^ Thai National Health Examination Survey (NHES V) conducted in 2014 reported that the overall prevalence of HT in Thailand was 25.6% and 23.9% in males and females, respectively. Almost half of adult Thais (44.7%) with HT were not even aware of their abnormal blood pressure [5]. Previous studies show that there are traditional and modifiable risk factors associated with HT including physical activities, smoking, alcohol consumption, stress, dietary behavior, and body mass index (BMI) [6]. Additionally, the recent studies reported that neck circumference (NC) was a simple anthropometric marker that correlated with free fatty acids, insulin resistance, very-low-density lipoprotein cholesterol (VLDL), and high BP level [7, 8]; moreover, another study recommended using NC as an additional screening tool for predicting cardiovascular risk [9].

However, in Thailand, when comparing access to health services in urban versus rural areas; there are more limitations for remote rural areas. Only limited information is available on factors potentially responsible for hypertension in remote rural communities. The required information is essential to focus on attenuating the problems. Improving awareness of hypertension and control will help to reduce any complications including metabolic syndrome and ASCVD. The objectives of this study were to determine HT and identify risk factors for HT among adults of a remote rural community in the central area of Thailand. Furthermore, we expect to find some valuable measurement of NC, which may be associated with hypertension in asymptomatic adults.

## Materials and methods

### Study design and population

This study was conducted in a rural community in the central area of Thailand, 180 km, from Bangkok Metropolitan: Phra Phloeng rural community in Sa Kaeo province. A cross-sectional study was performed in December 2016. A population-based total survey was used to collect the information from 3,700 participants. The participants were included those who had been living in 6 villages in Phrapleung. All of the participants were interviewed. Inclusion criteria were defined as adult people aged 20 years or more who resided in the community at the time the study was performed. Women who were pregnant more than 20 weeks or postpartum less than 6 weeks and those who were previously diagnosed with secondary hypertension were excluded.

The questionnaire was designed to record information from the participants, covered information on demographics, self-reported pre-existing comorbidities including diabetes mellitus (DM), dyslipidemia (DLP), smoking, alcohol consumption, physical activity based on the Global Physical Activity Questionnaire as recommended by WHO that recommend physical activity achieving at least 600 MET-minutes. DM and DLP were the patient’s comorbidities that were previously diagnosed by a health care professional or used those medications. Ex-smoking and Ex-drinker were defined as smoke-free and alcohol-free for 12 months. Non-smoker and Non- drinkers were patients who have never smoked and drunk alcohol in his or her lifetime.

1,084 adults were included in the study. The participants were recruited by having the researcher team visit their homes by door-knocking. The face-to-face interviews using standard questionnaires were used to obtain information from the participants.

### Clinical and laboratory parameters

Measurements were made using a standardized protocol. Bodyweight was determined without shoes and lightly clothed using an automatic electronic scale to the nearest 100 g. Height was measured without shoes to the nearest 0.5 centimeters. Waist circumference (WC) was measured after full expiration by using tape mid-way between the lowest rib and the top of the iliac crest. To measure NC, the participants were asked to extend their heads slightly and placed tape around the neck at the level of the Thyroid bone. Measure to be attached to the waist and neck fit, not too loose or too tight, and parallel with the ground. NC was categorized into two groups, i.e., normal (<37.5 cm in men) and <32.5 cm in women) and obesity (≥37.5 cm in men and ≥32.5 in women) [10]. BMI was calculated as body weight in kilogram divided by height in meters squared (m^2^) according to the criteria of the World Health Organization (WHO). We categorize the BMI as the WHO recommended cut-points for BMI categories in Asian populations as follows: 23–24.9 kg/m^2^, and ≥25 kg/m^2^ for overweight and obese [11]. The abdominal obesity as WC ≥ 90 cm for men and ≥ 80 cm for men women, according to the WHO-modified definitions for Asians [12].

HT was defined by The Seventh Report of the Joint National Committee on the Prevention, Detection, Evaluation, and treatment of High Blood Pressure (JNC’s seventh hypertension guidelines) as high BP (SBP≥140mmHg or DBP≥90mmHg) and/or use of antihypertensive medication [13]. BP measurements were performed using mercury sphygmomanometers. The operators were trained in the standardized technique. Participants were advised to sit quietly for at least five minutes in a chair, with feet on the floor and arms supported at heart level. Patients avoided caffeine, smoking, and exercise for at least 30 minutes before being measured. At least two measurements were made, and the average was recorded. Unawareness of HT was defined as not having self-reported any prior diagnosis of HT by a health care professional among the participants defined as having HT [14]. Treatment of HT was defined as using an antihypertensive drug prescribed by a healthcare professional.

### Statistical analysis

Data analyses were done using SPSS Statistics 22.0 and STATA/SE ver.11.0 program. We use descriptive statistical analysis to describe the prevalence, percentage, mean and SD, and analytic statistical analysis for associated factors by using univariate analysis with logistic regression analysis to determine the relationship between the exposures and outcome (hypertension) by presenting the magnitude of association with Crude odds ratio, at the 95% confidence intervals and p-value <0.05. Then, analyzing risk factors by controlling the potential confounding factors by multivariate analysis using multiple logistic regression analysis and presenting the relationship with Adjusted odds ratio. Confidence intervals, at the 95% level, were also reported for each adjusted odds ratio and p-value <0.05.

This study was approved by the Ethical Committee of the Royal Thai Army, Medical Department (approval number: 2559/67). The written informed consents were obtained from all participants in agreement with the WMA Declaration of Helsinki–Ethical principles for medical research involving human subjects.

## Results

### General characteristic of participants

The data was collected from the participants by using face-to-face interviews in December 2016. Table 1. Illustrates the demographic characteristics of participants. In total, there were 1,084 participants, 435 (40.1%) comprised males and 649 (59.9%) were females. Most of the participants were 50–59 years (28.7%). The average age of the population studied was 54.5 ± 13.1 years (range 20 to 97). The average NC of all participants was 34.5 ± 2.7 cm. The prevalence of HT among adults in a rural community was 27.7% (95% CI: 25.0–30.3). Of the 300 adults with HT, 66 participants (22%) were treated for HT while 164 participants (54.7%) were found within the unawareness HT category. To compare males and females, the prevalence of HT was 31.5% and 25.1%, respectively.

**Table 1:** General characteristics of participants enrolled into the study.

Characteristics	Non-hypertension	Hypertension	Total (n = 1,084)
	n (%)	n (%)	n (%)
**Gender**			
Male	298 (68.5)	137 (31.5)	435 (40.1)
Female	486 (74.9)	163 (25.1)	649 (59.9)
**Age (years) (mean ± SD)**	53.8 ± 13.4	56.5 ± 12.2	54.5 ± 13.1
**Age (years)**			
20–29	29 (85.3)	5(14.7)	34 (3.1)
30–39	72 (76.6)	22 (23.4)	94 (8.7)
40–49	207 (78.4)	57 (21.6)	264 (24.4)
50–59	219 (70.4)	92 (29.6)	311 (28.7)
60–69	155 (65.7)	81 (34.3)	236 (21.9)
70–79	82 (71.3)	33 (28.7)	115 (10.6)
≥80	20 (66.7)	10 (33.3)	30 (2.8)
**Education level**			
Illiterate	97 (76.4)	30 (23.6)	127 (11.7)
Primary school	84 (73.7)	30 (26.3)	114 (10.5)
Secondary school	581 (71.5)	232 (28.5)	813 (75)
University	22 (73.3)	8 (26.7)	30 (2.8)
**Occupations**			
Agriculture	405 (68.4)	187 (31.6)	592 (54.6)
Unemployed	112 (70.9)	46 (29.1)	158 (14.6)
Employee	50 (76.9)	15 (23.1)	65 (6)
Merchant	23 (65.7)	12 (34.3)	35 (3.2)
Officer	12 (92.3)	1 (7.7)	13 (1.2)
Others	182 (82.4)	39 (17.6)	221 (20.4)
**Smoking**			
Non-smoker	541 (74.5)	185 (25.5)	726 (67.0)
Ex-smoker	101 (67.8)	48 (32.2)	149 (13.7)
Smoker	142 (67.9)	67 (32.1)	209 (19.3)
**Alcohol drinking**			
Non-drinker	392 (73.5)	141 (26.5)	533 (49.2)
Ex-drinker	156 (71.9)	61 (28.1)	217 (20.0)
Alcoholic	236 (70.7)	98 (29.3)	334 (30.8)
**BMI (kg/m** ^**2**^ **) (mean ± SD)**	23.8 ± 3.9	25.3 ± 4.4	24.2 ± 4.1
**BMI (kg/m** ^**2**^ **)**			
<18.5	52 (74.3)	18 (25.7)	70 (6.5)
18.5–22.99	310 (80.7)	74 (19.3)	384 (35.4)
23–24.99	167 (71.7)	66 (28.3)	233 (21.7)
25–29.99	202 (67.1)	99 (32.9)	301 (27.8)
≥ 30	53 (55.2)	43 (44.8)	96 (8.9)
**Waist circumference (cm) (mean ± SD)**	83.3 ± 9.5	87.7 ± 10.9	84.5 ± 10.1
**Waist circumference (cm)**			
Normal (< 90 in male, < 80 in female)	402 (78.2)	112 (21.8)	514 (47.4)
Obesity (≥ 90 in male, ≥ 80 in female)	382 (67)	188 (33)	570 (52.6)
**Neck circumference (cm) (mean ± SD)**	34.3 ± 2.4	35.2 ± 3.2	34.5 ± 2.7
**Neck circumference (cm)**			
< 37.5 in male, < 32.5 in female	388 (75.8)	124 (24.2)	512 (47.2)
≥ 37.5 in male, ≥ 32.5 in female	396 (69.20)	176 (30.8)	572 (52.8)
**Comorbidities**			
Pre-existing diabetes mellitus	55 (53.9)	47 (46.1)	102 (9.41)
Pre-existing dyslipidemia	74 (54.4)	62 (45.6)	136 (12.55)

### Factors associated with hypertension

Table 2 illustrates univariate analysis for factors associated with HT. We found that females, old age (≥ 60 years old), BMI, WC, NC, occupation (agriculture, merchant, and unemployed), pre-existing diabetes mellitus, and pre-existing dyslipidemia were correlated with HT. Table 3 illustrate the models of multivariate analysis for factors associated with HT stratified by gender for the continuous and categorical variables of NC (continuous (model A) and categorical (model B). We found that NC both continuous and categorical variables are strongly significant with HT in all models.

**Table 2:** Univariate logistic regression analysis to assess relationships between associated risk factor and hypertension in the rural communities in Thailand.

	Crude Odds ratio	95% CI*	p-value
**Gender**			
Male	1.00		
Female	0.73	0.56–0.96	0.022
**Age (10 years)**	1.17	1.06–1.29	0.003
**Age**			
20–39	1.00		
40–59	1.31	0.83–2.10	0.256
≥ 60 years old	1.81	1.12–2.90	0.015
**Body Mass Index (kg/m** ^**2**^ **)**			
<23	1.00		
23–24.99	1.56	1.08–2.24	0.018
25–29.99	1.93	1.38–2.69	<0.001
≥30	3.19	2.01–5.07	<0.001
**Body Mass Index (kg/m**^**2**^**)** (continuous variable)	1.09	1.06–1.12	<0.001
**Waist circumference (cm)**			
Normal (< 90 in male, < 80 in female)	1.00		
obesity (≥ 90 in male, ≥ 80 in female)	1.77	1.35–2.32	<0.001
**Waist circumference (cm)** (continuous variable)	1.05	1.03–1.06	<0.001
**Neck circumference (cm)**			
< 37.5 in male, < 32.5 in female	1.00		
≥ 37.5 in male, ≥ 32.5 in female	1.39	1.06–1.82	0.020
**Neck circumference (cm)** (continuous variable)	1.11	1.05–1.16	<0.001
**Occupation**			
Others	1.00		
Agriculture	2.16	1.46–3.17	<0.001
Employee	1.40	0.71–2.74	0.327
Merchant	2.44	1.12–5.31	0.025
Officer	0.39	0.05–3.08	0.371
Unemployed	1.98	1.18–3.12	0.009
**Smoking**			
Non-smoker	1.00		
Ex-smoker	1.39	0.95–2.04	0.091
Smoker	1.38	0.99–1.93	0.060
**Alcohol drinking**			
Non-drinker	1.00		
Ex-drinker	1.09	0.76–1.55	0.643
Alcoholic	1.15	0.85–1.57	0.355
**Pre-existing diabetes mellitus**	2.18	1.44–3.32	<0.001
**Pre-existing dyslipidemia**	2.22	1.53–3.23	<0.001
**Activity**			
Not recommend (< 600 MET**-minutes per week)	1.00		
Recommend (≥ 600 MET-minutes per week)	0.85	0.64–1.14	0.279
**Family history of hypertension**	1.25	0.89–1.75	0.193

*CI: Confidence Interval;

**MET: Metabolic Equivalent.

**Table 3:** Multivariate logistic regression analysis to assess relationships between associated risk factor and hypertension stratify by gender (male, female and total).

	Male			Female			All		
Model A: Neck circumference as continuous variable	aOR*	95%CI**	p-value	aOR*	95%CI**	p-value	aOR*	95%CI**	p-value
Model A1									
Neck circumference (cm)	1.12	1.03–1.21	0.007	1.10	1.02–1.19	0.011	1.11	1.05–1.18	<0.001
Female							1.08	0.75–1.55	0.674
Model A2									
Neck circumference (cm)	1.13	1.04–1.23	0.004	1.10	1.03–1.20	0.009	1.12	1.06–1.19	<0.001
Female							1.13	0.79–1.62	0.784
Age (10 years)	1.01	0.99–1.03	0.152	1.02	1.00–1.03	0.030	1.02	1.00–1.03	0.011
Model A3									
Neck circumference (cm)	1.12	1.02–1.22	0.011	1.10	1.02–1.19	0.014	1.11	1.05–1.18	<0.001
Female							1.05	0.73–1.52	0.728
Age (10 years)	1.01	0.99–1.03	0.424	1.01	0.99–1.03	0.076	1.01	0.99–1.02	0.060
Pre-existing diabetes mellitus	2.81	1.24–6.38	0.013	1.99	1.00–3.11	0.047	2.06	1.30–3.27	0.002
Model A4									
Neck circumference (cm)	1.12	1.03–1.22	0.010	1.09	1.01–1.18	0.020	1.10	1.04–1.71	0.001
Female							1.01	0.69–1.46	0.957
Age (10 years)	1.01	0.99–1.03	0.405	1.01	0.99–1.02	0.164	1.01	0.99–1.02	0.105
Pre-existing diabetes mellitus	2.95	1.22–7.13	0.016	1.35	0.73–2.50	0.334	1.76	1.07–2.89	0.026
Pre-existing dyslipidemia	0.88	0.34–2.00	0.757	1.84	1.07–3.17	0.026	1.46	0.93–2.29	0.096
Model A5									
Neck circumference (cm)	1.12	1.03–1.22	0.009	1.09	1.01–1.18	0.020	1.10	1.04–1.71	0.001
Female							1.01	0.69–1.46	0.967
Age (10 years)	1.01	0.99–1.03	0.318	1.01	0.99–1.03	0.162	1.01	0.99–1.02	0.091
Pre-existing diabetes mellitus	3.01	1.24–7.31	0.015	1.35	0.73–2.51	0.335	1.76	1.07–2.89	0.027
Pre-existing dyslipidemia	0.83	0.36–1.91	0.659	1.84	1.07–3.17	0.026	1.44	0.99–2.27	0.106
Waist circumference (cm)	0.99	0.97–1.00	0.174	0.99	0.98–1.01	0.868	0.99	0.98–1.00	0.325
Model A6									
Neck circumference (cm)	1.13	1.04–1.24	0.006	1.10	0.01–1.19	0.023	1.11	1.05–1.78	0.001
Female							0.96	0.66–1.41	0.857
Age (10 years)	1.01	0.99–1.03	0.193	1.01	0.99–1.03	0.298	1.01	0.99–1.02	0.099
Pre-existing diabetes mellitus	2.72	1.13–6.61	0.026	1.47	0.79–2.77	0.220	1.83	1.10–3.03	0.019
Pre-existing dyslipidemia	0.81	0.35–1.86	0.621	1.86	1.07–3.22	0.026	1.43	0.91–2.25	0.123
Body Mass Index (kg/m^2^)	0.99	0.98–1.01	0.549	0.99	0.98–1.02	0.961	0.99	0.98–1.00	0.590
**Model B: Neck circumference as categorical variable**									
Model B1									
Neck circumference (cm)									
<37.5 in male, < 32.5 in female	1.00			1.00			1.00		
≥ 37.5 in male, ≥32.5 in female	2.90	1.74–4.83	<0.001	1.95	1.23–3.09	0.004	2.34	1.65–3.31	<0.001
Female							0.59	0.42–0.83	0.003
Model B2									
Neck circumference (cm)									
<37.5 in male, < 32.5 in female	1.00			1.00			1.00		
≥ 37.5 in male, ≥32.5 in female	3.07	1.82–5.19	<0.001	1.99	1.25–3.16	0.003	2.42	1.70–3.44	<0.001
Female							0.59	0.42–0.84	0.003
Age (10 years)	1.01	0.99–1.03	0.114	1.02	1.00–1.03	0.051	1.01	1.00–1.03	0.015
Model B3									
Neck circumference (cm)									
<37.5 in male, < 32.5 in female	1.00			1.00			1.00		
≥ 37.5 in male, ≥32.5 in female	2.72	1.59–4.66	<0.001	1.91	1.20–3.05	0.006	2.27	1.59–3.23	<0.001
Female							0.58	0.41–0.83	0.003
Age (10 years)	1.01	0.99–1.03	0.333	1.01	0.99–1.03	0.096	1.01	0.99–1.02	0.060
Pre-existing diabetes mellitus	2.53	1.12–5.74	0.026	1.54	0.89–2.67	0.120	1.81	1.15–2.84	0.010
Model B4									
Neck circumference (cm)									
<37.5 in male, < 32.5 in female	1.00			1.00			1.00		
≥ 37.5 in male, ≥32.5 in female	2.71	1.58–4.64	<0.001	1.82	1.14–2.91	0.012	2.20	1.54–3.14	<0.001
Female							0.57	0.40–0.81	0.002
Age (10 years)	1.01	0.98–1.03	0.347	1.01	0.99–1.03	0.212	1.01	0.99–1.02	0.115
Pre-existing diabetes mellitus	2.44	1.02–5.84	0.046	1.18	0.65–2.16	0.584	1.51	0.93–2.46	0.098
Pre-existing dyslipidemia	1.10	0.49–2.47	0.809	1.83	1.07–3.14	0.027	1.54	0.99–2.41	0.055
Model B5									
Neck circumference (cm)									
<37.5 in male, < 32.5 in female	1.00			1.00			1.00		
≥ 37.5 in male, ≥32.5 in female	1.95	1.02–3.74	0.042	1.86	1.12–3.10	0.017	1.93	1.30–2.89	0.001
Female							0.57	0.40–0.82	0.002
Age (10 years)	1.01	0.98–1.03	0.161	1.01	0.99–1.02	0.331	1.01	0.99–1.02	0.095
Pre-existing diabetes mellitus	2.33	0.91–5.94	0.077	1.48	0.79–2.75	0.219	1.71	1.02–2.85	0.040
Pre-existing dyslipidemia	0.97	0.42–2.23	0.950	1.64	0.94–2.86	0.080	1.38	0.87–2.19	0.165
Waist circumference (cm)									
Normal (< 90 in male, < 80 in female)	1.00			1.00			1.00		
obesity (≥ 90 in male, ≥ 80 in female)	1.10	0.93–1.34	0.346	1.17	0.85–1.39	0.470	1.12	0.90–1.23	0.305
Model B6									
Neck circumference (cm)									
<37.5 in male, < 32.5 in female	1.00			1.00			1.00		
≥ 37.5 in male, ≥32.5 in female	2.01	1.10–4.12	0.038	1.82	1.12–3.10	0.017	1.76	1.14–2.70	0.010
Female							0.55	0.38–0.79	0.001
Age (10 years)	1.01	0.99–1.03	0.257	1.01	0.99–1.03	0.292	1.01	0.99–1.02	0.090
Pre-existing diabetes mellitus	3.11	1.18–8.15	0.021	1.41	0.75–2.63	0.284	1.86	1.01–3.10	0.018
Pre-existing dyslipidemia	0.95	0.40–2.21	0.899	1.72	0.98–3.00	0.057	1.37	0.87–2.17	0.176
Body Mass Index (kg/m^2^)									
<23	1.00			1.00			1.00		
23–24.99	3.15	0.76–8.18	0.318	1.43	0.48–4.28	0.524	2.25	1.12–4.52	0.022
25–29.99	1.78	0.84–3.79	0.131	1.45	0.75–2.79	0.268	1.55	0.95–2.55	0.080
≥30	3.54	1.64–7.62	0.001	1.31	0.75–2.31	0.343	1.89	1.20–2.98	0.006

*aOR: adjusted odds ratio;

**CI: Confidence Interval;

Multivariate logistic regression analysis, Backward Wald method (Model A: neck circumference as continuous variable and Model B: neck circumference as categorical variable)

In model A, NC as the continuous variable was used in the analysis. After adjusting for neck circumference (continuous variable), gender, age, pre-existing diabetes mellitus, and pre-existing dyslipidemia (model A4), the adjusted odds ratio (aOR) along with the 95% confidence interval (95%CI) of association between NC and HT was 1.10 (1.04–1.71). Further, the WC or BMI was added for adjusting in models A5 (WC) and A6 (BMI), we found that NC and pre-existing diabetes mellitus were statistically significantly associated with HT. The aOR and 95%CI of NC and pre-existing diabetes mellitus in model 5 and model 6 were 1.10 (1.04–1.11), 1.76 (1.07–2.89), 1.11 (1.05–1.78), and 1.83 (1.10–3.03), respectively. In addition, sub-analysis stratified by gender was performed. Among male participants, NC and pre-existing diabetes mellitus were associated with HT and among female participants, NC and pre-existing dyslipidemia were associated with HT in model A4-6.

In model B, NC as the categorical variable was used in the analysis. After adjusting for neck circumference (categorical variable), gender, age, pre-existing diabetes mellitus, and pre-existing dyslipidemia (model B4), the adjusted odds ratio (aOR) along with the 95% confidence interval (95%CI) of association between NC (≥ 37.5 in males, ≥32.5 in females) and HT was 2.20 (1.53–3.14). Same as model A, the WC or BMI was added for adjusting in models B5 (WC) and B6 (BMI). We found that the factors associated with HT were include NC (aOR = 1.93 (1.30–2.89)), female (aOR = 0.57 (0.40–0.82)), and pre-existing diabetes mellitus (aOR = 1.71 (1.02–2.85)) in model B5 (WC) and NC (aOR = 1.76 (1.14–2.70)), female (aOR = 0.55 (0.38–0.79)), pre-existing diabetes mellitus (aOR = 1.86 (1.01–3.10)), BMI: 23–24.9 kg/m^2^ (aOR = 2.25 (1.12–4.52)), and BMI ≥ 30 kg/m^2^ (aOR = 1.89 (1.20–2.98)) in model B6 (BMI). Although Neck circumference is related to hypertension in all models regardless of adjusting WC or BMI, the correlation between higher neck circumference (≥ 37.5 cm. in male, ≥32.5 cm. in female) and HT tended to decrease after adjusting WC or BMI. Sub-analysis stratified by gender also performed in model B and NC was associated with HT in all models. In addition, pre-existing diabetes mellitus and BMI ≥30 kg/m^2^ were associated with HT among male participants (model B6).

## Discussion

The study aimed to determine the prevalence and associated factors of HT in a Thai remote rural community, especially higher neck circumference. The prevalence of HT in this study was 27.7%. To compare with the prevalence of HT in NHES V in 2014 [5], the results in the present study are relatively high. One potential explanation for this higher percentage is in this area, working-age adults were working temporarily in urban cities. Obviously, most of the population is older than 50 (63.8%). In addition, the Thai NHES V was conducted in urban and suburban areas, differing from the study in a remote rural community. We found that the prevalence of HT among adults in other rural areas in Asian countries differs from our finding in this study. Some countries like India [15] and Vietnam [6] indicated that the prevalence of HT was 25% and 20.8% respectively, while the studies in Myanmar [16] and China [17] show a higher prevalence of HT when compared with our report. The latest previous study of a Thai rural area in 2006 [18] reported that the prevalence of HT among adults in rural areas was 18.0%, which was lower than our findings. It might be because of a change of dietary habits, economic development, and lifestyle to be more urban.

Furthermore, we found that 54.7% of adults with HT were unaware of their high blood pressure. Similar to the other studies reported in Asia, the unawareness of HT was also higher than 50% among people at risk [19]. Compared to the unawareness HT in Thai NHES V [5] of 44.7%, the findings in this remote rural community were relatively high. This phenomenon can be explained by the difference of context in the area. Firstly, in the rural community, the people may have difficultly accessing the health care system; moreover, the people may have not enough health literacy, especially for HT [20]. Additionally, the low socioeconomic status in rural areas may also explain this situation [21].

Our study found that higher neck circumference, gender, and pre-existing diabetes mellitus are associated with HT. The prevalence of HT in females is lower than that in males. There were several studies [22, 23] reported that females tend to have lower rates of HT than males. However, the postmenopausal stage in which estrogen is depleted plays a major role in vasodilation resulting in increased blood pressure in elderly females [24]. Moreover, the previous study of Everett B et al. showed that females may be more aware of their diseases and therefore more likely to access the health care service when compared with males [22]. Furthermore, there are differences in RAS system receptors in gender which affect blood pressure. As angiotensin II type 2 receptor (AT2) has a role in cardioprotective effect, in general, there are more AT2 expressions in females than those in males, resulting from sex chromosome complement and estrogen [25].

In our study, the participants who have high neck circumference both continuous and categorical (≥ 37.5 in males, ≥ 32.5 in females) variables were more likely to have HT. Similar to a related study in the US, the Framingham Heart Study, which followed 2,732 peoples for 10 years (1995–2008) reported that higher neck circumference was associated with HT and all cardiometabolic risk factors [7]. A recent study in Thailand showed that high neck circumference was associated with an increase of blood pressure resulting in uncontrolled HT [8] The phenomenon can be explained by a significant correlation between NC and a pathogenic fatty deposit [7]. Moreover, the previous study by Koutsari et al. [26] described that upper–body obesity provides excessive adipose tissue lipolysis which leads to high oxidative stress, stimulates endothelial cell dysfunction and vascular injury, resulting in increased blood pressure. Additionally, the previous studies found that NC was related to obstructive sleep apnea (OSA), which was significantly associated with HT [27]. The mechanisms that influenced the increased risk for HT in OSA patients were pro-inflammatory effect, increased oxidative stress, and increase vascular stiffness.

WC and BMI are obviously used as a measure of obesity and risk assessment for CVD risk in clinical guidelines. Furthermore, several studies were found that waist circumference is significantly associated with increased blood pressure [28]. Although prior studies report that the cardio-metabolic risk can be predicted by WC and waist-to-hip ratio (WHR) [29], measuring is difficult due to the position to measure, fullness and clothing [30]. NC measurement is convenient; moreover, the measurement is constant and time-saving. The previous study [31] examined the association between NC and HT. It found that NC was strongly associated with HT, and the association became less magnitude of association after adjusting for BMI or NC. Moreover, the recent study suggests that the sensitivity, specificity, positive and negative predictive values of NC are better than WC in the marker of excessive fat tissue deposition which correlated with HT risk factors [32].

DM and HT usually co-exist in the patient. However, it is difficult to tell distinctly the mechanisms of DM that cause hypertension and mechanisms of hypertension that lead to DM because they are so similar, and it is difficult to clarify the initiative of each mechanism. DM can be the cause of hypertension due to several mechanisms including hyperinsulinemia, hyperglycemic crisis, and vascular dysfunction [33]. Hyperinsulinemia may lead to vascular injury and promote the vascular remodeling process resulting in vascular dysfunction [34]. Likewise, in hyperglycemia and hyperglycemic crisis, there are oxidative stress and inflammation which lead to cellular and endothelial dysfunction [34].

### Limitation of the study

One of the limitations of our study was it could not determine a temporal relationship because the study employed a cross-sectional design. Even though our study used standard measurement of HT, the masked HT may have resulted from the prevalence of HT in the study. Our study cannot be generalized to the whole country but may reflect challenges to patients residing in rural communities of Thailand. All exposures were self-reported and did not include blood biochemical data. Therefore, there might cause a recall bias. Nevertheless, this study used face-to-face interviews. So, this bias might also exist in this study. Due to the unavailable of data, we cannot investigate the effect of the anti-hypertensive drug on the relationship between NC and hypertension. We suggest the further study should take this issue into account by making an adjustment or analysis in a separate population of untreated and treated with the anti-hypertensive drug. The last limitation was that more than one-half of the participants were elderly working-age people immigrating to urban areas, causing the prevalence of high BP to be higher than it normally should be.

## Conclusions

In conclusion, almost one-third of participants in the remote rural community presented HT, and one-half of adults with HT were unaware of their condition. We identified potential risk factors for HT including being male, pre-exciting DM, especially and NC. The findings from this study confirmed that NC was associated with HT independent from other risk factors. Effective public health interventions including weight management, glycemic control, and suitable lipid profile should be provided to people in the rural community. Eventually, education concerning HT and its complications should be implemented in rural communities to attenuate HT and cardiovascular sequelae.

## References

[1] Who A. Global brief on hypertension. World Health Organization. 2013.

[2] Levy D, Larson MG, Vasan RS, Kannel WB, Ho KK. The progression from hypertension to congestive heart failure. Jama. 1996;275(20):1557–62. 8622246

[3] Dickinson CJ. Strokes and their relationship to hypertension. Curr Opin Nephrol Hypertens. 2003;12(1):91–6. doi: 10.1097/00041552-200301000-00015 12496672

[4] Worldwide trends in blood pressure from 1975 to 2015: a pooled analysis of 1479 population-based measurement studies with 19.1 million participants. Lancet (London, England). 2017;389(10064):37–55. doi: 10.1016/S0140-6736(16)31919-5 27863813 PMC5220163

[5] Institute HSR. Thai National Health Examination V (NHES V) 2014 [cited 2019 May 1]. https://www.hsri.or.th/researcher/research/new-release/detail/7711.

[6] Do HT, Geleijnse JM, Le MB, Kok FJ, Feskens EJ. National prevalence and associated risk factors of hypertension and prehypertension among Vietnamese adults. American journal of hypertension. 2014;28(1):89–97. doi: 10.1093/ajh/hpu092 24862960

[7] Preis SR, Massaro JM, Hoffmann U, D’Agostino RB Sr, Levy D, Robins SJ, et al. Neck circumference as a novel measure of cardiometabolic risk: the Framingham Heart study. The journal of clinical endocrinology & metabolism. 2010;95(8):3701–10. doi: 10.1210/jc.2009-1779 20484490 PMC2913042

[8] Meelab S, Bunupuradah I, Suttiruang J, Sakulrojanawong S, Thongkua N, Chantawiboonchai C, et al. Prevalence and associated factors of uncontrolled blood pressure among hypertensive patients in the rural communities in the central areas in Thailand: A cross-sectional study. PloS one. 2019;14(2):e0212572. doi: 10.1371/journal.pone.0212572 30779818 PMC6380583

[9] Zanuncio VV, Pessoa MC, Pereira PF, Longo GZ. Neck circumference, cardiometabolic risk, and Framingham risk score: Population-based study. Revista de Nutrição. 2017;30(6):771–81.

[10] Alzeidan R, Fayed A, Hersi AS, Elmorshedy H. Performance of neck circumference to predict obesity and metabolic syndrome among adult Saudis: a cross-sectional study. BMC obesity. 2019;6:13. doi: 10.1186/s40608-019-0235-7 30984406 PMC6442431

[11] Inoue S, Zimmet P, Caterson I, Chunming C, Ikeda Y, Khalid A, et al. The Asia-Pacific perspective: redefining obesity and its treatment. Sydney: Health Communications Australia Pty Ltd. 2000.

[12] Organization WH. Waist circumference and waist-hip ratio: report of a WHO expert consultation, Geneva, 8–11 December 2008. 2011.

[13] Chobanian AV, Bakris GL, Black HR, Cushman WC, Green LA, Izzo JL Jr., et al. The Seventh Report of the Joint National Committee on Prevention, Detection, Evaluation, and Treatment of High Blood Pressure: the JNC 7 report. Jama. 2003;289(19):2560–72. doi: 10.1001/jama.289.19.2560 12748199

[14] Gu D, Reynolds K, Wu X, Chen J, Duan X, Muntner P, et al. Prevalence, awareness, treatment, and control of hypertension in China. Hypertension. 2002;40(6):920–7. doi: 10.1161/01.hyp.0000040263.94619.d5 12468580

[15] Anchala R, Kannuri NK, Pant H, Khan H, Franco OH, Di Angelantonio E, et al. Hypertension in India: a systematic review and meta-analysis of prevalence, awareness, and control of hypertension. Journal of hypertension. 2014;32(6):1170. doi: 10.1097/HJH.0000000000000146 24621804 PMC4011565

[16] Htet AS, Bjertness MB, Oo WM, Kjøllesdal MK, Sherpa LY, Zaw KK, et al. Changes in prevalence, awareness, treatment and control of hypertension from 2004 to 2014 among 25-74-year-old citizens in the Yangon Region, Myanmar. BMC public health. 2017;17(1):847. doi: 10.1186/s12889-017-4870-y 29073891 PMC5659019

[17] Li J, Shi L, Li S, Xu L, Qin W, Wang H. Urban-rural disparities in hypertension prevalence, detection, and medication use among Chinese Adults from 1993 to 2011. International journal for equity in health. 2017;16(1):50. doi: 10.1186/s12939-017-0545-7 28288635 PMC5348878

[18] Howteerakul N, Suwannapong N, Sittilerd R, Rawdaree P. Health risk behaviours, awareness, treatment and control of hypertension among rural community people in Thailand. Asia-Pacific journal of public health. 2006;18(1):3–9. doi: 10.1177/10105395060180010201 16629432

[19] Son P, Quang N, Viet N, Khai P, Wall S, Weinehall L, et al. Prevalence, awareness, treatment and control of hypertension in Vietnam—results from a national survey. Journal of human hypertension. 2012;26(4):268. doi: 10.1038/jhh.2011.18 21368775

[20] Li W, Gu H, Teo KK, Bo J, Wang Y, Yang J, et al. Hypertension prevalence, awareness, treatment, and control in 115 rural and urban communities involving 47 000 people from China. Journal of hypertension. 2016;34(1):39–46. doi: 10.1097/HJH.0000000000000745 26630211

[21] Grotto I, Huerta M, Sharabi Y. Hypertension and socioeconomic status. Curr Opin Cardiol. 2008;23(4):335–9. doi: 10.1097/HCO.0b013e3283021c70 18520717

[22] Everett B, Zajacova A. Gender differences in hypertension and hypertension awareness among young adults. Biodemography and social biology. 2015;61(1):1–17. doi: 10.1080/19485565.2014.929488 25879259 PMC4896734

[23] Ghosh S, Mukhopadhyay S, Barik A. Sex differences in the risk profile of hypertension: a cross-sectional study. BMJ open. 2016;6(7):e010085. doi: 10.1136/bmjopen-2015-010085 27466234 PMC4964242

[24] Otsuka T, Takada H, Nishiyama Y, Kodani E, Saiki Y, Kato K, et al. Dyslipidemia and the Risk of Developing Hypertension in a Working-Age Male Population. Journal of the American Heart Association. 2016;5(3):e003053. doi: 10.1161/JAHA.115.003053 27016576 PMC4943276

[25] Gillis EE, Sullivan JC. Sex Differences in Hypertension: Recent Advances. Hypertension. 2016;68(6):1322–7. doi: 10.1161/HYPERTENSIONAHA.116.06602 27777357 PMC5159215

[26] Koutsari C, Jensen MD. Thematic review series: patient-oriented research. Free fatty acid metabolism in human obesity. Journal of Lipid Research. 2006;47(8):1643–50. doi: 10.1194/jlr.R600011-JLR200 16685078

[27] Gonzaga C, Bertolami A, Bertolami M, Amodeo C, Calhoun D. Obstructive sleep apnea, hypertension and cardiovascular diseases. Journal of human hypertension. 2015;29(12):705. doi: 10.1038/jhh.2015.15 25761667

[28] Momin M, Fan F, Li J, Jia J, Zhang L, Zhang Y, et al. Joint Effects of Body Mass Index and Waist Circumference on the Incidence of Hypertension in a Community-Based Chinese Population. Obesity facts. 2020;13(2):245–55. doi: 10.1159/000506689 32213776 PMC7250363

[29] de Koning L, Merchant AT, Pogue J, Anand SS. Waist circumference and waist-to-hip ratio as predictors of cardiovascular events: meta-regression analysis of prospective studies. European heart journal. 2007;28(7):850–6. doi: 10.1093/eurheartj/ehm026 17403720

[30] Namazi N, Larijani B, Surkan PJ, Azadbakht L. The association of neck circumference with risk of metabolic syndrome and its components in adults: A systematic review and meta-analysis. Nutrition, Metabolism and Cardiovascular Diseases. 2018;28(7):657–74. doi: 10.1016/j.numecd.2018.03.006 29779782

[31] Liang J, Wang Y, Dou L, Li H, Liu X, Qiu Q, et al. Neck circumference and prehypertension: the cardiometabolic risk in Chinese study. 2015;33(2):275. 10.1097/HJH.0000000000000396 PMC5325723 25545838

[32] Moradi S, Mohammadi H, Javaheri A, Ghavami A, Rouhani MH. Association between neck circumference and blood pressure: a systematic review and meta-analysis of observational studies. Hormone and metabolic research = Hormon-und Stoffwechselforschung = Hormones et metabolisme. 2019;51(8):495–502. doi: 10.1055/a-0957-3256 31408895

[33] Cheung BM, Li C. Diabetes and hypertension: is there a common metabolic pathway? Current atherosclerosis reports. 2012;14(2):160–6. doi: 10.1007/s11883-012-0227-2 22281657 PMC3314178

[34] Lastra G, Syed S, Kurukulasuriya LR, Manrique C, Sowers JR. Type 2 diabetes mellitus and hypertension: an update. Endocrinology and Metabolism Clinics. 2014;43(1):103–22. doi: 10.1016/j.ecl.2013.09.005 24582094 PMC3942662

